# Supplementary material for: Association between oral microbiome and seven types of cancers in East Asian population: a two-sample Mendelian randomization analysis
Source: Front Mol Biosci. 2023 Nov 21;10:1327893. doi: 10.3389/fmolb.2023.1327893 (PMC10702768; doi:10.3389/fmolb.2023.1327893)
Supplement: Supplementary file 1 [file DataSheet1.ZIP › Supplementary Materials/MR plots of saliva/saliva═╝/ovarian cancer/pheno.2340_to_ovarian cancer_leave_one_out.pdf]

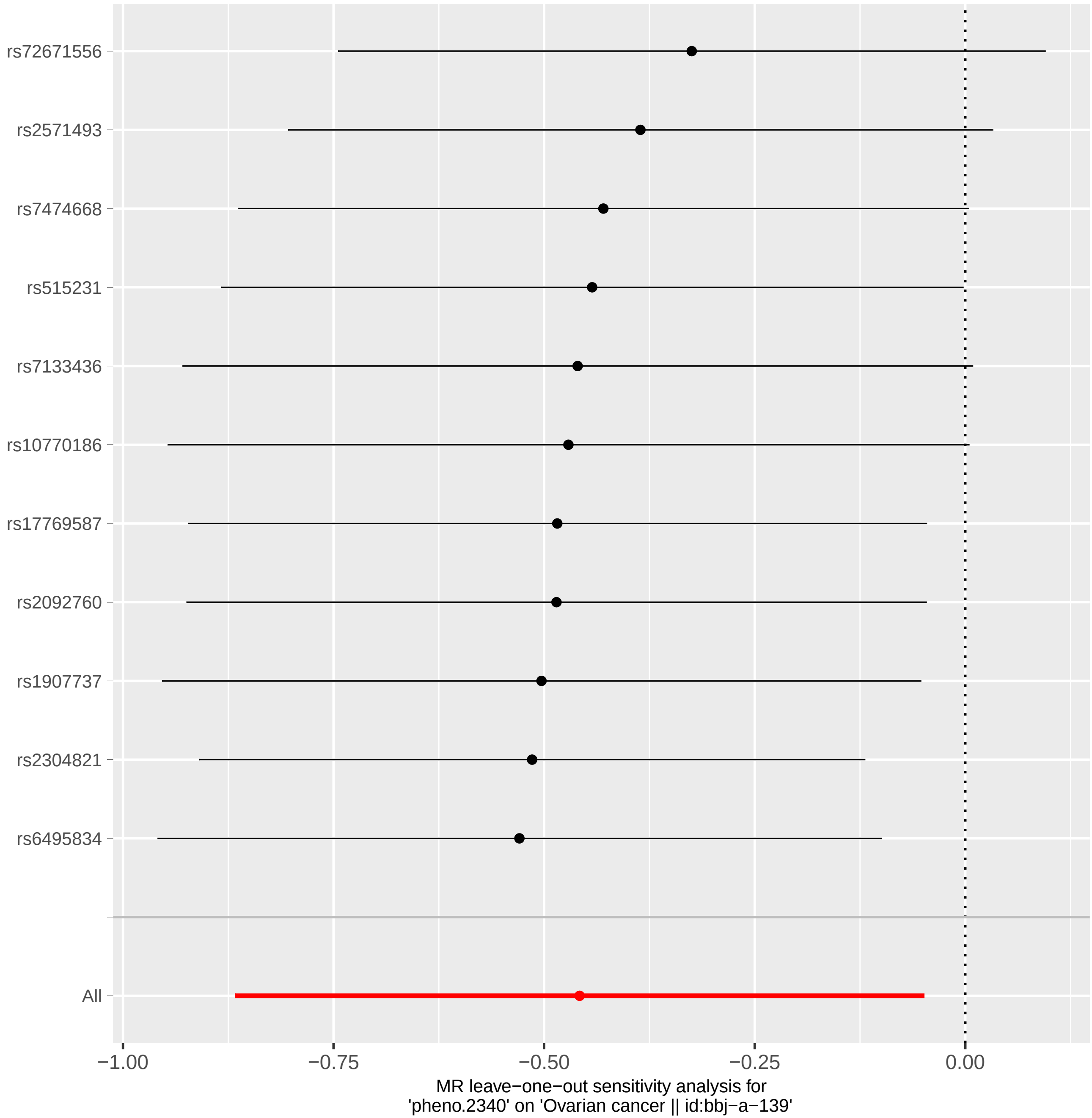
rs72671556
rs2571493
rs7474668
rs515231
rs7133436
rs10770186
rs17769587
rs2092760
rs1907737
rs2304821
rs6495834
All
−1.00
−0.75
−0.50
−0.25
0.00
MR leave−one−out sensitivity analysis for
'pheno.2340' on 'Ovarian cancer || id:bbj−a−139'
